# Models of Hfq interactions with small non-coding RNA in Gram-negative and Gram-positive bacteria

**DOI:** 10.3389/fcimb.2023.1282258

**Published:** 2023-10-24

**Authors:** Derrick Watkins, Dev Arya

**Affiliations:** ^1^Department of Math and Science, University of Tennessee Southern, Pulaski, TN, United States; ^2^Laboratory for Medicinal Chemistry, Department of Chemistry, Clemson University, Clemson, SC, United States

**Keywords:** RNA, bacteria, *E. coli*, Hfq, protein

## Abstract

Hfq is required by many Gram-negative bacteria to chaperone the interaction between small non-coding RNA (sRNA) and mRNA to facilitate annealing. Conversely and despite the presence of Hfq in many Gram-positive bacteria, sRNAs in Gram-positive bacteria bind the mRNA target independent of Hfq. Details provided by the Hfq structures from both Gram-negative and Gram-positive bacteria have demonstrated that despite a conserved global structure of the protein, variations of residues on the binding surfaces of Hfq results in the recognition of different RNA sequences as well as the ability of Hfq to facilitate the annealing of the sRNA to the mRNA target. Additionally, a subset of Gram-negative bacteria has an extended C-terminal Domain (CTD) that has been shown to affect the stability of the Hfq hexamer and increase the rate of release of the annealed sRNA-mRNA product. Here we review the structures of Hfq and biochemical data that have defined the interactions of the Gram-negative and Gram-positive homologues to highlight the similarities and differences in the interactions with RNA. These interactions provided a deeper understanding of the how Hfq functions to facilitate the annealing of sRNA-mRNA, the selectivity of the interactions with RNA, and the role of the CTD of Hfq in the interactions with sRNA.

## Introduction

Small non-coding RNAs (sRNA) have been shown to regulate a variety of different responses in bacteria by acting as a posttranscriptional regulator of translation and are often associated with adaptive stress response, including antibiotic resistance (Repoila and Darfeuille, 2009; Waters and Storz, 2009; Chakravarty and Masse, 2019; Watkins and Arya, 2019; Diallo and Provost, 2020; Avican et al., 2021; Ponath et al., 2022). The most common mechanism of mRNA regulation by sRNA is accomplished by Watson-Crick base pairing of the sRNA to the ribosomal binding site of the mRNA resulting in the silencing of the mRNA (Thisted et al., 1994; Franch et al., 1999; Brunel et al., 2002). While the silencing of the mRNA by sRNAs occurs in both Gram-negative and Gram-positive bacteria, current data suggests that there is greater use of the chaperone protein, Hfq, by Gram-negative bacteria to facilitate the interaction between the sRNA and mRNA compared to Gram-positive bacteria (Jousselin et al., 2009; Nielsen et al., 2010; Santiago-Frangos et al., 2016).

Hfq was first identified in *Escherichia coli* (Ec) as an essential Host factor for the replication of bacteriophage Qβ RNA (Franze de Fernandez et al., 1968; Franze de Fernandez et al., 1972), and is found in ~50% of all bacteria (Sun et al., 2002). Hfq is a pleiotropic protein and has been shown to interact with multiple types of nucleic acids including both RNA and DNA as well as various proteins (Brennan and Link, 2007; Orans et al., 2020; Cossa et al., 2022; Dendooven et al., 2023). One function of Hfq is in wild-type regulation of FtsZ abundance and cell division (Takada99).

The interactions of Hfq with RNA typically acts to suppress protein expression, but has also been shown in some instances to enhance the expression of the mRNA (Repoila and Darfeuille, 2009). Hfq has been shown to bind sRNA and mRNA independently of each other and can act to protect the mRNAs or signal the degradation of mRNA even in the absence of sRNA (Mohanty et al., 2004). However, the most common function associated with Hfq is the chaperoning of the interaction between sRNA and mRNA. This function of Hfq appears to be restricted to the homologues in Gram-negative bacteria. While the need for Hfq facilitation has been linked to the GC content of the RNA, the length of the “seed region” of the RNA, and the difference in *cis*-encoded and *trans-*encoded sRNAs (Jousselin et al., 2009), the ability of the Hfq to facilitate the interactions between mRNA and sRNA is associated with the differences in the structures of the Hfq homologues as described below.

## The structure of Hfq

**Figure 1:**
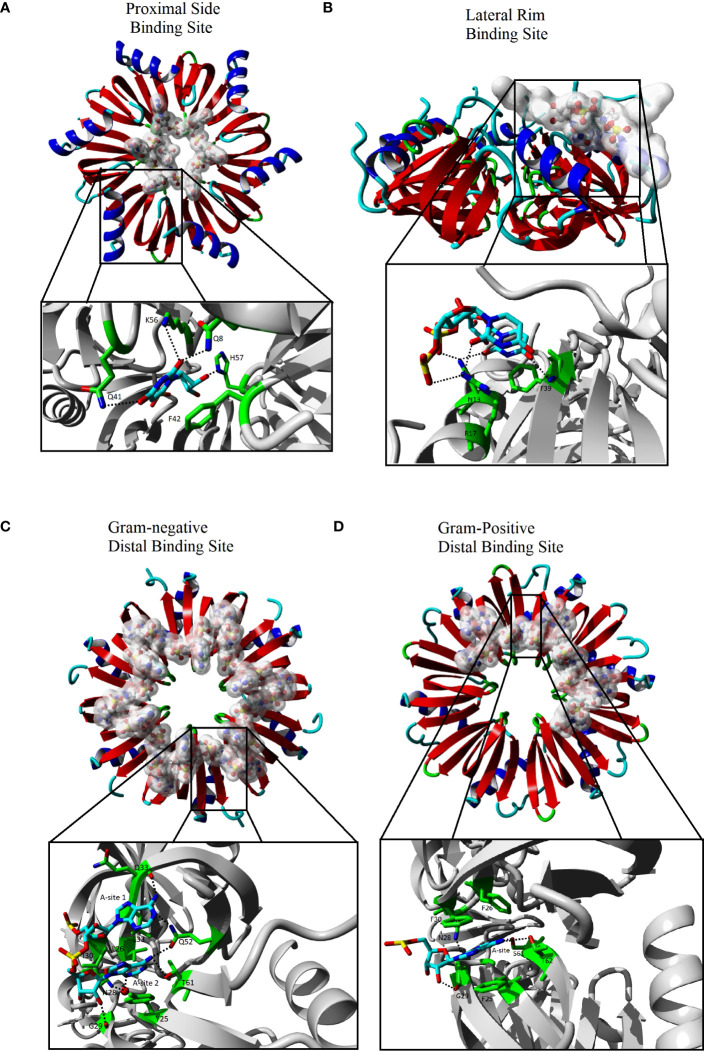
The interactions of Hfq with RNA. The Hfq core domain is composed of six monomers represented as red and blue ribbons. Each binding site recognizes a different sequence of RNA that is represented as sticks that are highlighted with a white cloud in the far view (C-F upper). The expanded view (C-F lower black box) shows the Hfq residues (green sticks) involved in base specific interactions with the RNA (cyan sticks). The expanded views have been rotated relative to global view for clarity. Images generated using Yasara (Krieger and Vriend, 2014; Krieger and Vriend, 2015) using Hfq species and PDBs of **(A)** Sa Hfq PDBID: 2ylc **(B)** Ec Hfq PDBID: 4v2s **(C)** Ec Hfq PDBID: 3gib **(D)** Bs Hfq PDBID: 3hsb. Some RNA residues were removed from the original models for clarity.

The global structure of Hfq is a hexameric protein made up of six monomers of the Sm/Lsm family. Each subunit consists of 5 β-sheets and 1 N-terminal α−helix. All current structures of Hfq show that the global structure of the hexamer is conserved among both Gram-negative and Gram-positive bacteria and produces a “donut” shaped structure with two sides that each contain an independent RNA binding site (Schumacher et al., 2002; Sauter et al., 2003; Nikulin et al., 2005; Nielsen et al., 2007; Boggild et al., 2009; Someya et al., 2012; Kovach et al., 2014). The convex side of Hfq, with the solvent exposed α-helices of each monomer, is defined as the proximal side, and the distal side is defined as the convex surface in which most of the β-sheets solvent exposure occurs (**Figure 1**). A third binding site, referred to as the lateral rim, has also been identified on outer lateral surface of the Hfq core furthest from the central pore, and is made up by residues of the C-terminus of the α-helix (Robinson et al., 2014). The three binding sites allow Hfq to bind two different strands of RNA simultaneously which is fundamental to the ability of Hfq to act as a chaperone of the annealing of sRNA and mRNA (Park et al., 2021). Additionally, Hfq has a C-terminal Domain (CTD) following the Lsm core that is highly variable in length and sequence. The CTD appears to be highly flexible and typically no residues of the CTD are observed in the structures of Hfq. In fact, the CTD of Hfq was only partially observed in three of the structures of the protein (Beich-Frandsen et al., 2011; Dimastrogiovanni et al., 2014; Santiago-Frangos et al., 2019). The CTD likely has multiple functions in Hfq and has been shown to be involved in the formation of amyloids involved in the packing of DNA as well as the interactions with the cell membrane (Cossa et al., 2022; Turbant et al., 2022). However, the CTD appears to play an important, species-specific role in the chaperone activity of Hfq as described below.

### Proximal side

**Figure 2:**
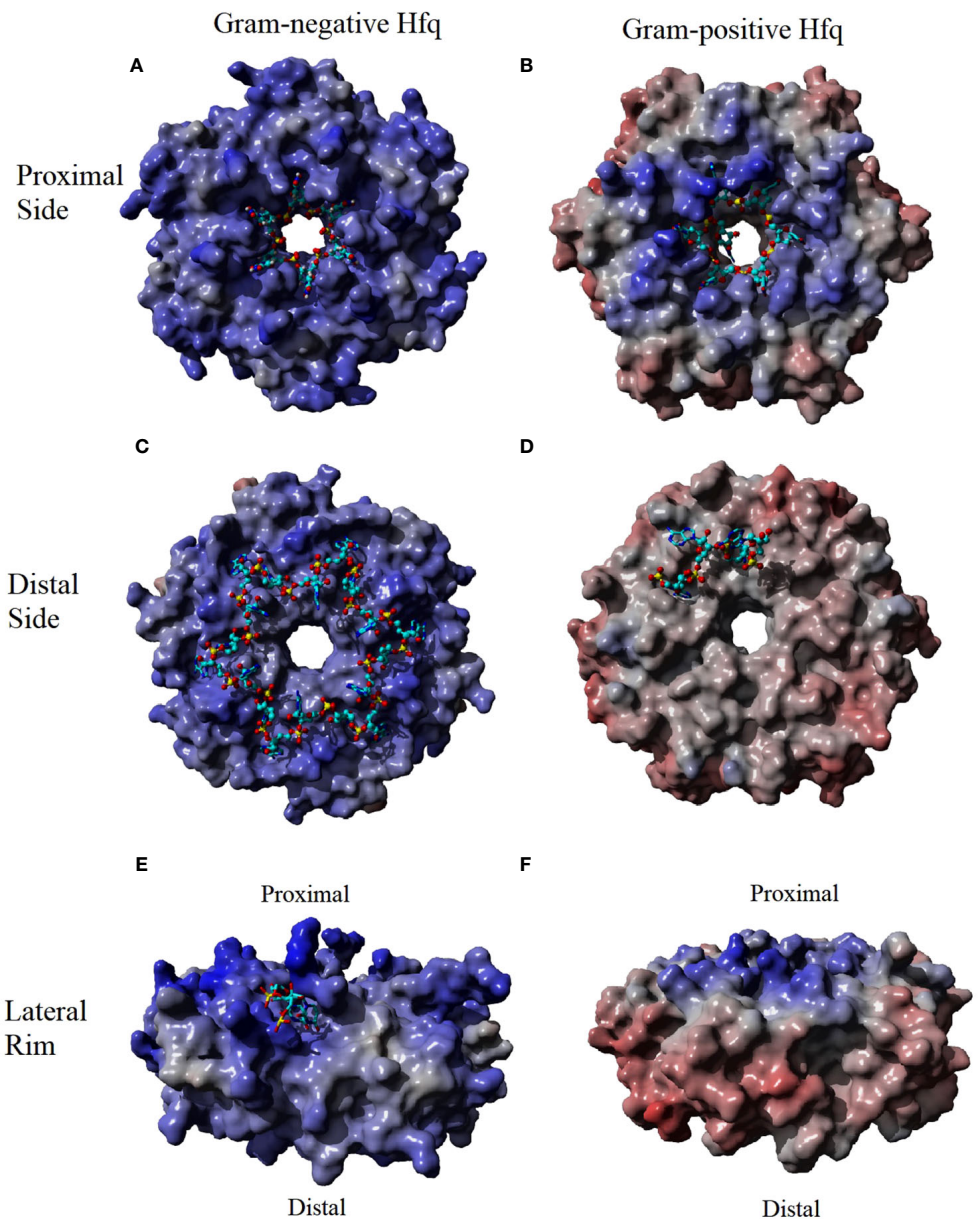
The Electrostatic Surface Potential (ESP) of Hfq-RNA binding surfaces. The binding surfaces of Hfq in Gram-negative bacteria have a greater positive potential (indicated as a blue surface) than the Gram-positive homologue. The RNA (cyan sticks) interacts with the positive potential of these surface. The greatest difference in the ESP occurs at the rim (compare **E**, **F**) where the potential is positive in Gram-negative and negative in Gram-positive (red surface). ESP were generated using Yasara (Krieger and Vriend, 2014; Krieger and Vriend, 2015) using an Amber96 forcefield (Kollman P et al., 1997) and a particle mesh Ewald simulation (Essman U et al., 1995) using Hfq species and PDBs of **(A)** Sa Hfq PDBID: 2ylc **(B)** Ec Hfq PDBID: 4v2s **(C)** Ec Hfq PDBID: 3gib **(D)** St Hfq PDBID: 3qsu. Some RNA residues were removed from the original models for clarity.

The proximal side of Hfq is highly conserved between homologues of Hfq and has been shown to bind single strand RNA close to the central pore of Hfq on this side (**Figure 1A**) (Schumacher et al., 2002; Brennan and Link, 2007; Sauer and Weichenrieder, 2011; Wang et al., 2013; Robinson et al., 2014). All structures with RNA bound to the proximal side have the single strand RNA bound to the positive electrostatic surface of Hfq in a circular pattern about the pore with one nucleotide bound to each monomer of Hfq (**Figures 2A, B**). The binding site on the proximal face accommodates uridine or adenine bases with a preference for U-rich sequences with a free 3’OH common in many sRNAs (Sauer and Weichenrieder, 2011). The base bound in the proximal side binding site stacks with a conserved aromatic residue (Ec, *Salmonella typhimurium* (Sa) and *Bacillus subtilis* (Bs) residue F42, *Staphylococcus aureus* (St) Y42). Hfq residues make a base specific contact through a conserved Q8 that recognizes an O2 of uridine or O6 of adenine, a conserved K56 residue and an amine group at position 41 (Ec and Sa Q41, Bs K41 and St N41). A conserved H57 (Ec and Sa numbering) interaction with O2’ of the ribose likely ensures a preference for RNA over DNA binding. The proximal side binding site appears to be a general binding site of all Hfq homologues and binds to single strand A/U rich RNA sequences and may participate in the rearrangement of secondary structures of the RNA (Sauer et al., 2012).

### Distal side

The various homologues of Hfq have only minor differences in the aligned primary sequences of the protein (Sonnleitner et al., 2002). However, the crystal structure of Hfq bound to RNA, along with biochemical data, have shown the changes in the sequence result in differences to distal side RNA binding sites between Gram-positive and Gram-negative bacteria (Link et al., 2009; Horstmann et al., 2012; Someya et al., 2012; Wang et al., 2013; Robinson et al., 2014). The distal binding site of Gram-negative and Gram-positive is typically responsible for recognizing the 5’-polyA sequence of mRNA, but the distal site has also been shown to act as a second binding site for some sRNAs in Gram-negative Hfq homologues as discussed below (Schu et al., 2015). The distal side of Gram-positive is less electrostatically positive compared to the Gram-negative homologue of Hfq (**Figures 2C, D**). In Gram-negative bacteria, the distal side contains six RNA binding sites with each site recognizing the sequence (A-A-N)_n_ (Robinson et al., 2014). Interaction between the protein and the RNA involves two different subunits of Hfq with adenine specific contacts between the Q52 and the backbone of Q33 and a hydrophobic interaction with L32 in the same subunit of Hfq create the first A binding site of the (A-A-N)_n_ motif of the RNA in Gram-negative homologues (**Figure 1C**). Residue Q52 bridges the first and second A-site making contacts to the adenine in both sites. Additional base specific contacts occur with T61 of the adjacent subunit of Hfq, and the water mediated N28 in the second A-site. Additional hydrophobic interactions are observed between the adenine in the second A-site with L26, I30 and Y25 of the adjacent subunit. The N-site has no base specificity, as no contacts between the base of the N-site and the protein have been observed. The N-site appears to bridge adjacent binding sites and may act as an entry point of the adenines (Link et al., 2009; Wang et al., 2013; Robinson et al., 2014).

Similar to Gram-negative bacteria, Gram-positive bacteria have six binding sites, with each binding site located at the interface of two subunits of Hfq. However, an inserted amino acid in the loop between β-strands 3 and 4 and a small number of seemingly conservative variations, particularly I30 to an aromatic (F30 in Bs, Y30 in St), in Gram-positive Hfq results in a slight shifting of the first A-site and a loss of the second A-site (**Figure 1D**) (Horstmann et al., 2012; Someya et al., 2012). These changes result in a distal recognition sequence of (A-L)_n_ where the L indicates a linker between the each site and, similar to the N-site of Gram-negative Hfq, can be any nucleotide. The change in the distal binding site from an (A-A-N) to an (A-L) recognition not only alters the specificity of the sequence bound on the distal side, but also reduces the maximum number of bases bound from 18 nucleotides by Gram-negative Hfq to 12 by the Gram-positive Hfq.

### Lateral rim

The difference in the electrostatic surface potential of the lateral rim of Hfq between Gram-negative and Gram-positive homologues plays a significant role in the difference in the annealing function of Hfq homologues (Zheng et al., 2016). The surface of the rim of Gram-negative Hfq homologues is significantly more electropositive than those of Gram-positive (**Figures 2E, F**). Sequence alignment of the rim has shown that the Hfq of Gram-negative bacteria are enriched with arginine residues in this area compared to Gram-positive bacteria, and Ec residues R16, R17, and R19, referred to as the arginine patch, are highly conserved in Gram-negative bacteria, but less so in Gram-positive. The rate of RNA annealing increases as the number of arginine residues increases on the rim of Hfq and is independent to changes to the proximal and distal side binding sites (Sauer et al., 2012; Panja et al., 2013; Zhang et al., 2013; Cai et al., 2022). The structure of Hfq bound to a full length RhyC sRNA identified an additional RNA binding site that has specificity for single strand UU dinucleotides on the proximal face of the rim (**Figure 1B**) (Dimastrogiovanni et al., 2014). Additional contacts of the rim involve non-specific phosphate interactions in contact with the stem region of the RNA (Dimastrogiovanni et al., 2014). Combined with biochemical data the rim region has been implemented in the melting of the stem regions of RNA that contains the complementary seed sequences of the sRNA and the target mRNA (Panja et al., 2013). The RNA rearrangement exposes the single strand of each complementary sequence, allowing the annealing of the two strands and the formation of the sRNA-mRNA duplex (Sauer et al., 2012; Zheng et al., 2016). It has been proposed that the greater GC content of Gram-negative bacteria requires the positive arginine patch at the rim of Hfq to facilitate the rearrangement of the RNA, while the lower GC content of Gram-positive bacteria results in less stable stem regions that can rearrange independent of Hfq. This observation is supported by the lack of arginines in St homologues of Hfq and the Hfq independent annealing of sRNA to mRNA in St bacteria.

### C-terminal domain

The CTD of Hfq varies greatly not just between the Gram-positive bacteria where it is largely absent or severely truncated, but also within different species of Gram-negative bacteria in which the length of the CTD varies greatly (Sonnleitner et al., 2002; Sun et al., 2002). While the length of the CTD varies, key characteristics of the CTD have been identified in most Gram-negative bacteria. The highly conserved P64 is considered the end of the core domain and defines the start of the CTD. Residue R66 (*E. coli* numbering) is also highly conserved but has been shown to pack against the lateral edge of the core (Santiago-Frangos et al., 2017). The middle of the CTD has the greatest variability in both length and sequence and appears to be species specific. The final six residues define the tail of the CTD and contain acidic residues with a consensus sequence of DSEETE (Arluison et al., 2004; Santiago-Frangos et al., 2017).

## Model of interaction

Two models of the Hfq facilitated sRNA-mRNA annealing in Gram-negative bacteria have been proposed based on the differences observed in the sRNA that is involved in the interactions with Hfq. Two different classes of sRNA, Class I and Class II, have been identified based on these differences in the interaction with Hfq and the recognition sequence of the sRNA (Schu et al., 2015). Both classes of sRNA interact with Hfq at two different binding surfaces. Class I of sRNA appears to be the most common and interacts with the proximal side of Hfq with the U-rich 3’ Rho-independent terminator of the sRNA. The second binding site of Class I sRNA involves the rim of Hfq at the UU binding motif. The mRNA targets of Class I sRNAs bind to the distal side binding site via an AAN motif present on the mRNA. The formation of the sRNA-Hfq-mRNA ternary complex results in the annealing of the sRNA with the mRNA seed sequence at the rim. Following the annealing the sRNA and mRNA are both degraded by RNase activity (Schu et al., 2015).

Class II sRNA binds with the same binding motif to the proximal side of Hfq as Class I but the second binding site of the sRNA is on the distal side of Hfq with an AAN motif, as opposed to the rim. The mRNA targets of Class II sRNAs are unable to bind to the distal side of Hfq even if they contain an AAN motif and must therefore bind to the rim of Hfq at the UU binding site. The formation of the sRNA-Hfq-mRNA complex again results in the annealing of the sRNA with the mRNA sequence. However, in the case of Class II sRNA, the mRNA is degraded, but the sRNA often remains bound to Hfq for multiple rounds of mRNA binding (Schu et al., 2015). As a consequence of the different modes of interactions between Class I and Class II sRNAs with Hfq, the concentration Class I sRNAs are diminished with each interaction with their target and do not accumulate in the cell. Therefore, Class I sRNAs are self-regulated. However, Class II sRNAs can remain in the cell unless “deactivated” by other cellular mechanisms. As a result, initial studies have indicated that Class I sRNAs act as response element while Class II sRNA act as silencers of cell function (Figueroa-Bossi et al., 2009; Moon and Gottesman, 2009; Overgaard et al., 2009; Thomason et al., 2012; Hoe et al., 2013).

An additional consequence of the difference in the interaction between Class I and Class II sRNAs is the role of the CTD of the Hfq in the regulation of the process. Early work into the role function of the CTD appeared conflicting, with some work indicating that the CTD played no role in the Hfq interaction with sRNA, while other work indicates that the CTD is required for the proper binding and annealing of sRNA to the mRNA target (Sonnleitner et al., 2002; Sonnleitner et al., 2004; Vecerek et al., 2008; Olsen et al., 2010; Updegrove and Wartell, 2011; Salim et al., 2012). While differences in experimental methods may have been partially responsible for the conflicts, later findings have shown that the conflicts likely result from the differences in the function of the CTD that depends on the type of sRNA bound to Hfq, and involves a competitive interaction between the CTD and the RNA with the positive arginine patch on the rim of Hfq (Salim et al., 2012; Santiago-Frangos et al., 2016; Santiago-Frangos et al., 2017; Sarni et al., 2022).

The facilitation of annealing of Hfq for Class I and Class II sRNA in Ec is initiated by the sRNA and mRNA binding to Hfq. Both RNAs potentially undergo a rearrangement of stem regions exposing the seed sequence at the lateral rim region of Hfq. The exposure of the single strand seed sequence of the sRNA and mRNA following the rearrangement of the stem regions allows the annealing of the sRNA-mRNA at the rim of Hfq (Hwang et al., 2011; Sauer et al., 2012; Malecka and Woodson, 2021; Cai et al., 2022). Class I sRNA binding, annealing and release appears to occur independently of the CTD. The binding and annealing of Class II sRNAs also occur independent of the CTD, but the release of the annealed sRNA-mRNA product requires or is enhanced by the CTD (Kavita et al., 2022). The double strand product has a lower affinity for the rim of Hfq and is released from Hfq as the sRNA-mRNA hybrid. Unlike Class I sRNA, however, the rate of release of Class II sRNA-mRNA relies on the presence of the acidic tail CTD to displace the RNA duplex (Santiago-Frangos et al., 2016; Santiago-Frangos et al., 2017).

The CTD also plays an important role in discriminating between Class I and Class II binding of Hfq. In the presence of the CTD, Class II sRNAs bind preferentially to Hfq over Class I. However, the removal of the CTD removes the preference for Class II sRNA (Santiago-Frangos et al., 2016). It has been shown that the CTD does not contact the RNA, does not stabilize the proximal or distal RNA-Hfq interaction, and does not speed up the annealing process. Instead, the acidic tail of Hfq competes with the RNA for the basic patch of the rim. Therefore, the binding of the Class I sRNAs would be in competition with the CTD for the basic patches of the rim while Class II sRNAs would not face competition for the binding sites on the distal face of Hfq, Thus the role of the CTD of Hfq sets up a hierarchy for sRNA binding by reducing the apparent affinity of Class I sRNAs and giving rise to preferential binding of Class II sRNAs (Schu et al., 2015; Santiago-Frangos et al., 2016; Santiago-Frangos et al., 2017; Kavita et al., 2022).

Recent studies examining the stability of the Hfq using mass spectroscopy combined with the observations described above has led to a more comprehensive model of the chaperone activity of Hfq (Sarni et al., 2022). The CTD stabilizes the association of the monomers of the hexameric toroid structure of Hfq through both intra and inter-monomer interactions between the acidic tail of the CTD and the basic patch of the rim (Sarni et al., 2022). The displacement of the CTD results in a less stable structure, and the disruption of one of the CTD interactions cascades to disrupt the network of interactions. The disruption of the interaction can be stabilized by the binding of the sRNA. However, only sRNAs that properly interact with Hfq are able to effectively stabilize the structure. Therefore, RNAs that form favorable contacts with Hfq offset the loss of stability of displacing the CTD contact with the core domain of Hfq. The coordination of the binding of the RNA and the CTD at the rim of Hfq allows the CTD to screen the RNA interactions and prevent non-specific binding of RNAs to Hfq. Additionally, because Hfq has six different interactions between the CTD and core (one per monomer) the RNA can progressively displace the CTDs from the core. The progressive type interactions would therefore allow Hfq to sample different regions of the RNA for contacts or allow the binding of different RNAs sequentially without effecting the binding of another RNA molecule (Sarni et al., 2022).

## Conclusion

There are multiple differences in the binding surfaces of Hfq between Gram-positive bacteria compared to Gram-negative bacteria. The difference in the requirement of Hfq between the two classes of bacteria likely results in the origins of the sRNA in the different bacteria. The sRNA identified in Gram-positive bacteria are predominantly *cis*-encoded that are perfectly or highly complementary to the mRNA target. Gram-negative bacteria contain more *trans*-encoded sRNAs that have limited complementarity to the mRNA target (Peer and Margalit, 2014). Therefore, Gram-negative bacteria would benefit from Hfq that could chaperone the interaction of sRNA with its mRNA target, and it has been hypothesized that the evolution of Hfq has coincided with the evolution of *trans*-encoded sRNAs (Peer and Margalit, 2014). The co-evolution of sRNAs and Hfq has also been proposed to explain the greater importance of the CTD of Hfq in γ-proteobacteria. It will be interesting to see if newly discovered sRNA follow a similar trend of *cis* and *trans*-encoded sRNAs to support this hypothesis.

## Author contributions

DA: Writing – review & editing. DW: Writing – original draft.
